# Recent Advances in Oral Peptide or Protein-Based Drug Liposomes

**DOI:** 10.3390/ph15091072

**Published:** 2022-08-28

**Authors:** Jian Cui, Zhiwei Wen, Wei Zhang, Wei Wu

**Affiliations:** School of Pharmacy, Guilin Medical University, Guilin 541199, China

**Keywords:** polypeptide and protein drugs, oral administration, oral bioavailability, drug delivery system, liposomes

## Abstract

The high physiology and low toxicity of therapeutic peptides and proteins have made them a hot spot for drug development in recent years. However, their poor oral bioavailability and unstable metabolism make their clinical application difficult. The bilayer membrane of liposomes provides protection for the drug within the compartment, and their high biocompatibility makes the drug more easily absorbed by the body. However, phospholipids—which form the membranes—are subjected to various digestive enzymes and mucosal adhesion in the digestive tract and disintegrate before absorption. Improvements in the composition of liposomes or modifying their surface can enhance the stability of the liposomes in the gastrointestinal tract. This article reviews the basic strategies for liposome preparation and surface modification that promote the oral administration of therapeutic polypeptides.

## 1. Introduction

Peptides and proteins have been used with great success in treating cancer, genetic diseases, inflammation and diabetes. The activity and specificity of peptides cannot be imitated by small molecule drugs. Moreover, their complex spatial conformation and fragile tertiary structure have a high susceptibility to being broken by the harsh physiological environment of the gastrointestinal tract, resulting in their extremely low bioavailability. Currently, only oligopeptides or cyclic peptides are available for oral use, such as the FDA-approved macrocyclic drugs Linaclotide, for irritable bowel syndrome and Lutetium Lu 177 for gastroenteropancreatic neuroendocrine tumors [1].

To further expand the number of oral peptides, it is expected that improvements will be made in the oral absorption of the peptides via their formulation, and some promising therapeutic peptides have already entered the clinical phase such as oral delivery of Octreotide using Peptelligence technology, oral semaglutide based on Eligen technology, etc. [2]. However, there are still many promising therapeutic peptides in need of the design of novel delivery systems for their oral administration, such as GLP-1, Lactotransferrin, etc. Several oral drug delivery strategies have been developed to improve the bioavailability of therapeutic peptides—for example, self-emulsifying drug delivery systems (SEDDS) [3], solid lipid nanoparticles (SLN) [4], liposomes, and microgels [5].

Liposomes are spherical structures with phospholipids as their main component, and their biological properties are similar to cell membranes—making them ideal carriers for orally administered drugs. However, the stability of liposomes is poor due to the presence of digestive enzymes and extreme pH changes in the gastrointestinal tract. The use of liposomes as a carrier system for oral drug delivery faces great challenges. With the continuous development of nanotechnology, the oral delivery of liposomes has once again become a research hotspot, as shown in Table 1.

## 2. Properties of Protein and Peptide Drugs

Understanding the physicochemical properties of peptides provides the basis for the rational design and development of optimized formulation systems. Fogg et al. [21] reported that the P_app_ of therapeutic peptides smaller than 1400 Da has a significant negative correlation with molecular weight. The apparent permeability coefficients (P_app_) are used to reflect the permeability of drugs to cell membranes. Besides this, smaller peptides are easily captured by the internal aqueous phase in liposomes. Similarly, the charge and hydrophobicity of therapeutic peptides leads to their adsorption being altered, which can affect their retention in and release from nanoparticles.

The natural structure of proteins is susceptible to being changed by pH, ionic composition, temperature, or digestive enzymes. The use of unnatural amino acids (e.g., D-α, Nα-alkylated, Cα-substituted, β- and γ-amino acids) or amide bond mimetics (e.g., thioamides, azapeptides, 1,4 disubstituted 1,2,3-triazoles) that anchor peptide backbone-specific sites to form rigid structures can reduce the susceptibility of peptides to enzymatic degradation [22]. Cyclization reduces the polarity of the peptide molecule itself by eliminating the end groups, and is also a strategy for peptides to combat the harsh external environment [23].

## 3. Phospholipid Materials Suitable for the Oral Administration of Liposomes

The physicochemical properties of phospholipids represent a precondition for the prepared formulations. So far, a variety of lipids—including dipalmitoyl-phosphatidylcholine (DPPC), distearoyl-phosphatidylcholine (DSPC)—have been utilized to encapsulate therapeutic proteins such as insulin and salmon calcitonin (sCT) [24]. However, oral administration requires phospholipids that are not susceptible to hydrolysis and oxidation to enhance the rigidity of the lipid bilayer. Some specific lipids such as diether or tetraether lipids can maintain structural integrity at extreme pHs [25]. Amphiphilic polymers with properties similar to phospholipids such as polyoxyethylene alkyl ethers can be used to form more stable vesicle niosomes for oral administration. Bilayers composed of surfactant (non-ionic) and cholesterol called niosomes have lower toxicity and a more stable structure than conventional liposomes [26]. The novel bolalipids contain lateral alkyl chains of different lengths in the 1- and 32-positions of the long membrane-spanning C32 alkyl chain, which acts as a stabilizer of the liposome. It shows greater stability in phosphate buffer solution and in simulated gastric juice; the release of calcineurin encapsulated in Borealis liposomes is reduced by 50% in simulated gastric juice compared to normal liposomes [27].

## 4. Preparation Methods for Polypeptide Liposomes

The manufacture and production of peptide-liposomes face many challenges; the preparation process greatly affects the encapsulation of peptides and proteins by liposomes. The retention, protection, and release of bioactive proteins from vesicles are regulated by changing the sign and charge density of the phospholipid polar groups, as well as the pH and ionic strength of the dispersion medium [28,29]. The difference in charge density on the surface of liposomes (∆σ) before and after protein adsorption can be calculated by the following equation:

∆σ = σ_A_ − σ_B_ = 2eXZ_p_/A_L_ (1)

where X is the extent of protein adsorption (moles per mole of lipid), A_L_ is the average surface area of the phospholipid molecules, and Z_p_ is the effective charge on the protein.

It has been shown that adsorption is mainly dependent on the electrostatic interactions on the surface of the substance [30]. Coincidentally, Jacques-Philippe Colletier et al. revealed that at pH 8.5, the amphiphilicity phospholipid POPC showed a higher encapsulation rate of negatively charged acetylcholinesterase (AChE). Adding DOGS-NTA-Ni lipid induced a stronger interaction between the lipid bilayer and the enzyme, which leaded to a further increase in the encapsulation rate [31].

The pH and ionic strength of the dispersion medium also need to be considered; pHs further away from the PI significantly increase the trypsin encapsulation rate. Similarly, a lower ionic strength decreases protein solubility and facilitates interactions between proteins and lipid bilayers, which makes phospholipids more susceptible to capturing drug proteins [32].

Peptides can interact non-covalently with non-ionic surfactants through hydrogen bonding; hydrophobic interactions form water-insoluble complexes called peptide-surfactant complexes (PSC). Since the formation of hydrophobic ion pairs (HIP) increases the lipophilicity of peptide and protein drugs, they can be solubilized in the lipophilic phase of lipid-based nanocarriers to improve the drug encapsulation rate [33,34].

**Figure 1:**
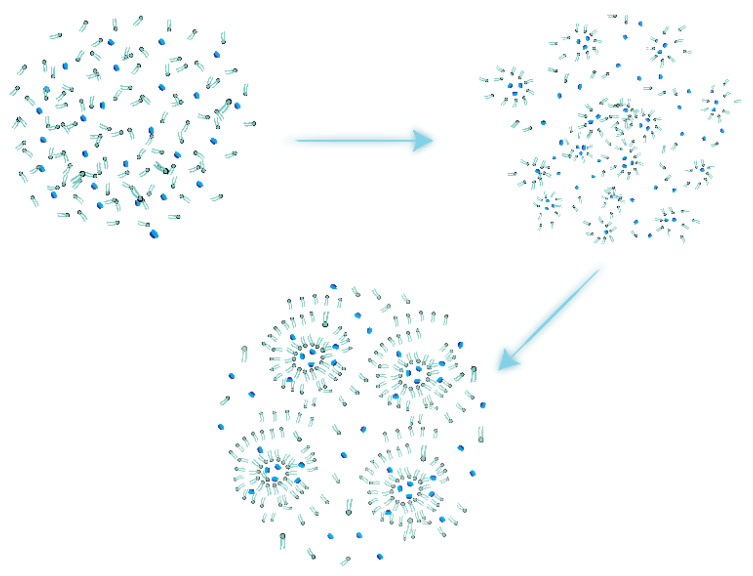
Liposome formation process.

In addition, temperature, high pressure, non-aqueous solvents, pH, ionic strength, and shear force during preparation affect protein stability [35]. Some different preparation methods can effectively avoid these adverse effects. Figure 1 shows the general liposome formation process. 

Freeze–Thaw Cycling (FTC) performed to prepare liposomes and to encapsulate proteins occurs in two steps: Empty lipid vesicles are prepared by thin-film hydrophoresis. Then, liposome suspensions are mixed with protein solutions and subjected to freeze–thaw cycles in liquid nitrogen (−196 °C) and a water bath (65 °C). Protein-loaded liposomes form while the liposome membranes fragment in the liquid nitrogen and reform in the water bath. Finally, the liposomes are extruded with a liposome extruder. The encapsulation efficiency of uncoated liposomes has previously been found to be 69%; the particle size was 174.8 ± 0.9 nm with a PDI of 0.19 ± 0.01 [7].

The microfluidic hydrodynamic focusing (MHF) method was first proposed by Jahn et al. [11]. Typically, in small microfluidic channels with diameters of up to 500 μm, gradient diffusion and local dilution of the organic phase in the aqueous phase of the laminar flow allows phospholipids to self-assemble into liposomes. By adjusting the flow rate ratio between the aqueous and the organic phases, Zehua Liu et al. improved the encapsulation of rhIns (recombinant human insulin) to 91 ± 4% and stabilized the particle size at 144 ± 23 nm. However, the use of organic solvents can lead to the partial inactivation of proteins and the deep penetration of organic solvents into the lipid bilayer, which may alter the mechanical and physical properties of the membrane [11].

**Figure 2:**
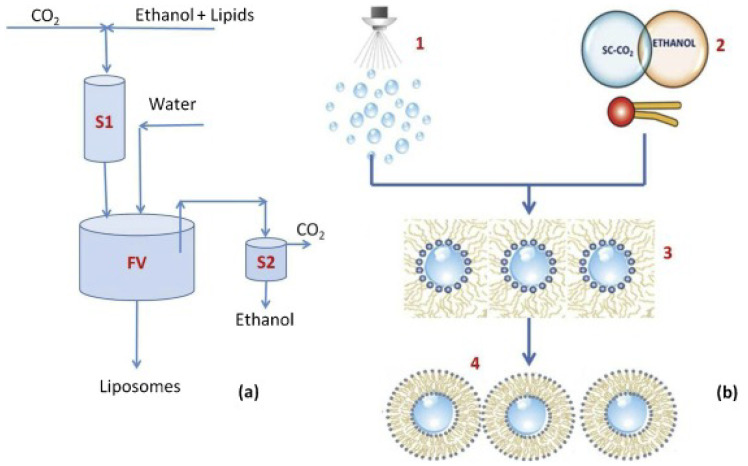
Supercritical-assisted liposome formation; SuperLip process layout (**a**) and a sketch of the phenomena occurring in it (**b**), adapted from Trucillo et al. [36].

Supercritical carbon dioxide as a co-solvent assists in encapsulating bovine serum albumin successfully and maintains its biological activity; encapsulation rates range from 92% to 98%. The solution containing the bovine serum is sprayed into a carbon dioxide supercritical fluid with dissolved lipids. Due to the extremely low surface tension of the dispersion medium, a lipid layer forms rapidly around the atomized droplets. Subsequently, the water/CO_2_ emulsions are mixed with aqueous solution to form liposomes [8]. Figure 2 shows the process of supercritical fluid-assisted liposome formation. Supercritical carbon dioxide produces an antisolvent effect when mixed with organic solvents containing dissolved phospholipids and drugs and induces the formation of precursor liposomes; Gang Yang et al. successfully prepared bile salt-liposomes encapsulated with silymarin (SM) with an average particle size of 160.50 nm and obtained an encapsulation rate of 91.38% [9]. Liposomes for oral application have been successfully prepared using supercritical fluid; they also show excellent encapsulation of proteins. Therefore, supercritical fluid can be used as a reference process for the development of oral liposomes of polypeptide and protein drugs.

The encapsulation rate and particle size are important parameters in the selection of an appropriate preparation method, and it is also important to avoid the destruction of protein by organic solvents as much as possible. The above process has its own advantages for protein encapsulation; most of them have a high encapsulation rate and relatively stable particle size compared with traditional methods.

## 5. Stability Strategy

Oral carriers are subjected to extreme pH changes (from an acidic environment in the stomach to a neutral or alkaline environment in the intestine) and the combined adverse effects of digestive enzymes (such as phospholipase, pancreatic lipase, and cholesterol esterase) and bile salts [37]. These conditions will affect the stability of liposomes. For instance, both phospholipases C and D can cleave the phosphorus oxygen bond of phosphate ester in phosphatidylcholine molecule to produce diacylglycerol or phosphatidylic acid. Diacylglycerol can cause large-scale lipid rearrangement and phase transition, resulting in changes in membrane thickness and the decomposition of liposomes [38].

**Figure 3:**
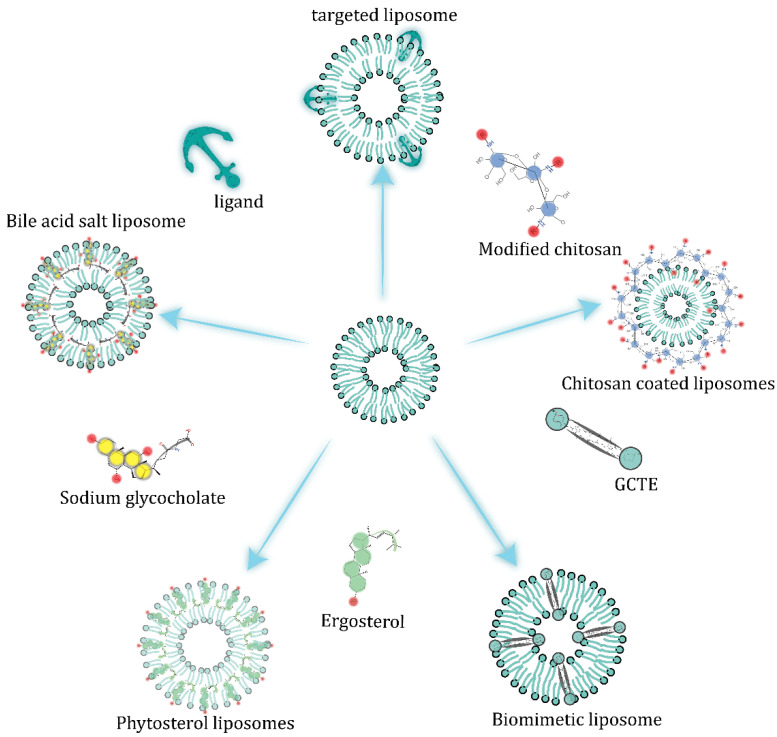
Liposome modification strategy.

Liposomes can resist the malignant physiological environment of the gastrointestinal tract through a variety of surface modifications, as shown in the Figure 3. More details are presented in Table 2.

### 5.1. Alteration of Liposome Membrane Composition

Glycorylcaldityl tetraether (GCTE) has a rigid structure that is resistant to hydrolysis and oxidation. Embedding into the phospholipid bilayer can stabilize the lipid membrane and make it resistant to gastric acid and trypsin A2 [39]. In a study by Schulze et al., Myrcludex B—which specifically aggregates in the liver [40]—can be encapsulated in liposomes containing 5% GCTE. The results showed that after oral administration to Wistar rats, the GCTE-liposome significantly enhanced the uptake of iodine-131-labeled Myrcludex B by the liver, which is approximately three times that of normal liposomes [25].

Some phytosterols have a similar structure to cholesterol and have stronger van der Waals interactions with the acyl chains of DPPC, making the liposomes resistant to the physiological environment of gastrointestinal tract (GIT); lipid bilayers with anionic phospholipids introduced and ergometrine embedded show excellent stability. A previous experiment has shown that free insulin is almost degraded within 15 min, while insulin encapsulated in sterol liposomes can still be retained at more than 70% after 4 h in simulated intestinal fluid. The highest transport efficiency of Er-Lip has also been observed in experiments on monolayer Caco-2 cell transport [41].

### 5.2. Embedded Bile Salts

Bile salts embedded in the lipid bilayer slow down the emulsifying effect of endogenous bile on the carrier. In a study by Niu et al., liposomes containing sodium glycopyrrolate (SGC) were prepared by reverse-phase evaporation, using insulin as a model drug. The results showed that the bioavailability of the insulin encapsulated by SGC-Liposome was about 8.5% and 11.0% in nondiabetic and diabetic rats, respectively. After oral administration in a gavage experiment in Wistar rats, a maximum 63% decrease in blood glucose was produced at 10 h after oral administration and returned to normal about at 20 h [42].

### 5.3. Surface-Coating Strategy

Surface modifications can significantly improve the stability of liposomes in the intestine by forming a contact barrier between the liposomal phospholipids and enzymes [43]. Liposomes coated with chitosan-thioglycolic acid polymers can enhance surface adhesion and permeability and inhibit lipids from degrading. The stability of liposomes modified with different molecular weights (77 KD and 150 KD) of chitosan-thioglycolic acid and the chitosan-thioglycolic acid 6-mercaptonicotinamide-conjugate (CS-TGA150-MNA) in simulated gastric fluid (SGF) and simulated intestinal fluid (SIF) has been tested. Liposomes have good stability in SGF and CS-TGA-modified liposomes have a slower release rate in SIF, demonstrating the resistance of CS-TGA coating to trypsin [44]. 

Hydrophilic silica nanoparticles on the surface of liposomes can form an interfacial layer to delay the release of drugs and to retard lipolysis by digestive enzymes. Liposomes containing insulin were prepared by TFH, concentrated by centrifugation and mixed with a gradient concentration of silica nanoparticles in ultrapure aqueous solution to produce silica-coated liposomes. In SIF, the lipolysis rate of the silica-coated liposomes was significantly reduced. In SGF, the hydrophilic silica nanoparticles delayed the release of insulin and enhanced the stability of the liposomes [45].

### 5.4. Diversified Dosage Forms

Alginate has a unique gel formation property in the presence of multivalent cations; mixing with liposome suspension containing bee venom, drop in calcium chloride solution to subsequently form calcium alginate gel beads loaded with bee venom liposomes, and coat the surface with Eudragit S100 to resist erosion of the nanoparticles by the gastric acid and digestive enzymes. In vitro release results have shown a negligible release of bee venom at pH 1.2, while bee venom release was triggered at pH 6.8 and pH 7.4. Gamma scintillation studies showed that ^99m^Tc-MIBI-labeled bee venom had a mean small intestinal transit time of 3.5 ± 0.5 h. The drug was released at the colon 4 h after administration, showing the stability of the drug in gastric and intestinal fluids [46].

As previously described, chitosan-coated liposomes (InsLip-CHT) encapsulated with insulin have been prepared by microfluidic techniques. Furthermore, Ins@MPs have been obtained by the double-emulsion microfluidic method; the nanoparticles were encapsulated in a double emulsion containing hydroxypropyl (HPMCAS-MF) as a way to improve the stability of the nanoparticles in the gastrointestinal tract. Ins@MPs showed responsive release in the simulated intestinal fluid (SIF, pH 6.8) and almost no release in SGF (pH 1.2). Caco-2 and HT29-MTX cell lines were used to assess the intestinal permeability of Ins@MPs. A much higher P_app_ of Ins@MPs than free insulin (P_app_ of 2.27 × 10^−5^ cm∙s^−1^) and a concomitant decrease in transepithelial electrical resistance (TEER) were observed. Functioning as a fluorescent probe, 4-(4-dihexadecylaminostyryl)-N-methylpyridinium iodide (DiA) has been encapsulated in chitosan liposomes (DiALip-CHT), accelerating their internalization by cells [11]. 

**Table 2:** The composition of liposomes and the enzymatic stability of both the encapsulated peptide/protein and the phospholipids.

API	Phospholipid	Strategy to Protect Liposomes from the Damage of GIT	Properties	EE (%) ± SD	MD (nm) ± SD	Zeta (mv) ± SD	Ref
Myrcludex B	EPC	GCTE, which is resistant to hydrolysis and oxidation, was embedded in the phospholipid bilayer	At least 7% of the initial dose of Myrcludex B was absorbed, with a 3.5-fold increase in oral effectiveness	65.7 ± 2.9	140.7 ± 4.3	−4.2 ± 0.5	[25]
rhINS	SPC DPPG Chol	Phytosterols with stronger interactions with phospholipids were used instead of cholesterol	After 4 h in SGF, Er-lip retained more than 70% of the insulin; the plasma glucose level could be reduced to about 60% of the initial value and kept low for 8 h	30 ± 2.0	157.1 ± 0.4	−60.5 ± 9.8	[41]
rhINS	SPC	GCA was able to reduce the degradation of liposomes in GIT and promote the internalization of lipid particles	High oral bioavailability of 11.0%, with a mild and lasting hypoglycemic effect	35 ± 2.1	358 ± 28.0	-	[42]
Calcitonin	PC DSPG Chol	Surface-modified CPPs and TMC promoted the cellular uptake of liposomes	Effectively enhanced the oral absorption of calcitonin	80 ± 2.0	118 ± 18.0	−27.1 ± 5.8	[47]
FID	DPPC DPPE-MCC	Chitosan coating with thiol group modification enhanced the adhesion and permeability of liposomes and inhibited the degradation of lipid membranes by enzymes	P_app_ was 2.8–3.4 times stronger than the initial value	-	702.6 ± 138.0	8.62 ± 1.4	[44]
Insulin	DPPC	Silica coating isolated liposomes from digestive enzymes	Silica coating was able to reduce the lipolysis rate and continuously release the drug for up to 8 h	70.0	297 ± 0.4	−15 ± 4.0	[45]
Bee venom	PC	Liposomes were encapsulated into Eudragit S100-coated calcium alginate gel microspheres to slow the drug leakage of liposomes at non-specific sites	Liposomes completed drug release at the colon and maintained structural integrity in Git	95.36 ± 0.3	2.05 ± 0.7 mm	-	[46]
rhINS	E-PC	Liposomes with chitosan coating were encapsulated into double extrusion by “two-step” microfluidic technology	Exhibited the characteristics of pH-responsive release and accelerated the intracellular internalization of encapsulated insulin	91 ± 4.0%	19 ± 1.0 μm	-	[11]

DPPE-MCC, 1,2-dipalmitoyl-sn-glycero-3-phosphoethanolamine-N-[4-(p-maleimidomethyl) cyclohexane-carboxamide]; FID, fluorescein isothiocyanate-dextran; rhINS, Recombinant human insulin; GCA, glycocholic acid; CS–TGA, chitosan–thioglycolic acid; CS–TGA–MNA, the chitosan-thioglycolic acid 6-mercaptonicotinamide-conjugate; CPPs, cell penetrating peptides; TMC, N-trimethyl chitosan chloride; DPPE-MCC, 1,2-dipalmitoyl-sn-glycero-3-phosphoethanolamine-N-[4-(p-maleimidomethyl) cyclohexane-carboxamide]; Er-lip, Ergosterol modified liposomes; E-PC, Egg-phosphatidylcholine.

## 6. Receptor-Mediated Transportation across Enterocytes

**Figure 4:**
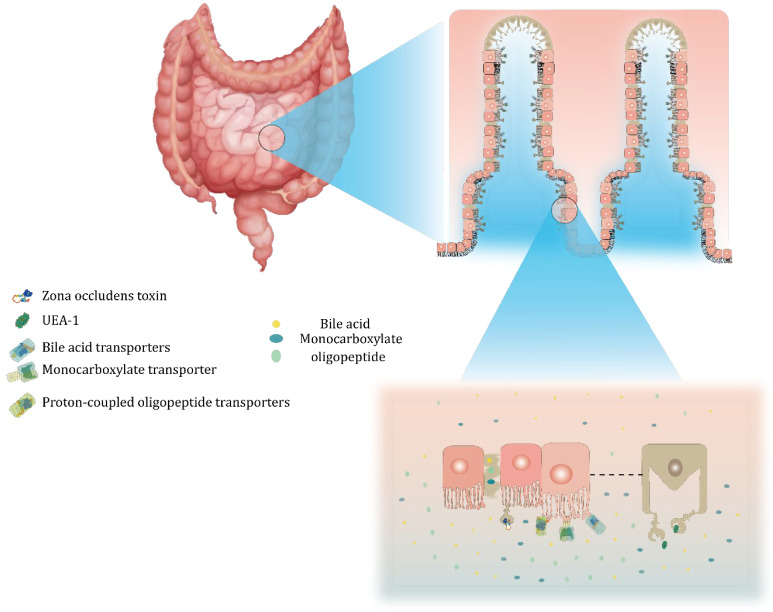
Intestinal absorption mechanism.

Typically, peptide and protein drugs can enter the systemic circulation from the intestinal lumen via the transcellular pathway (through cells) and the paracellular pathway (between cells) as well as via endocytosis, as shown in Figure 4.

Liposome surface modification with Fc domain-binding peptide (FcBP) to target neonatal Fc receptors (FcRns) increases liposome endocytosis at the apical surface of the intestinal epithelium as well as exocytosis on the basolateral side. Yu, et al. prepared insulin liposomes modified with FcBP using thin-film hydrophorasis. Caco-2 cell uptake assays showed that endocytosis was maximal at pH 6.0, while the exocytosis of FcBP-Lip was significantly elevated at pH 7.4. In addition, INS-FcBP-Lip produced a significant hypoglycemic effect, with a maximum reduction of 47.87% in the initial blood glucose level [20].

There are abundant folate receptors on intestinal epithelial cells, so combining folate and liposomes can improve the effective uptake of drugs. For example, Yazdi et al. prepared folate-modified insulin-loaded liposomes; in Caco-2 cell uptake assays, 125I-labeled insulin uptake was increased 1.2–1.5-fold in folic acid-coupled liposomes and their bioavailability was increased up to 19.08% [48].

In addition, bile salts embedded in liposome bilayers promote the internalization and absorption of particles in the small intestine through bile acid transporters. Fluorescence imaging has shown that fluorescently labeled insulin encapsulated in SGC-liposomes has the strongest fluorescence and the longest duration in GIT. In monolayer Caco-2 cell transport experiments, the transepithelial electrical resistance (TEER) was significantly reduced in the presence of SGC-liposomes, showing effective paracellular permeability and enhancing the uptake of small intestinal epithelial cells [49].

As previously described, CS-TGA150-MNA coating can enhance the stability of liposomes; similarly, promotion of the cellular internalization of liposomes was also observed. According to an in vivo small intestine transit assay, liposomes with this coating have the highest P_app_—which was approximately 4.2-times higher than that of the blank control—and the CS-TGA coating decreased the transepithelial electrical resistance (TEER) values. Thiomers indirectly inhibit protein tyrosine phosphatase (PTP) and thus open tight intercellular junctions, resulting in enhanced uptake of liposomes [44].

## 7. Small Intestine-Lymphatic Circulation Prevents the First-Pass Effect

The liver is the largest reticuloendothelial system (RES) organ; nearly all (∼85%) nanoparticles will be accumulated in the liver mononuclear phagocyte system (MPS) [50]. Their powerful metabolic capacity makes nanoparticles that have experienced gastrointestinal erosion unable to react. Therefore, it is necessary to seek a hepatic bypass pathway into the systemic circulation to avoid the damage of the first pass effect to the active pharmaceutical ingredient (API). Intestinal lymphatic transport is considered an alternative drug delivery strategy. Targeting microfold cells (M cells) or forming chylomicrons (CMs) can mediate the entry of APIs into the systemic circulation through gut-associated lymphoid tissue [51]. For example, both Cholera toxin B subunit (CTB)—which is non-toxic to humans—and Ulex europaeus 1 (UEA-1) can specifically target M cells [52,53]. FITC-labeled surface-modified UEA-1 liposomes show stronger adhesion to Peyer’s patch M-cells. In vivo experiments show that oral administration of hepatitis B surface antigen (HBsAg) encapsulated in UEA-1-modified liposomes induced the highest sIgA levels in the intestine. As the main antibody type present in the GIT, the upregulation of sIgA content suggests that targeting M cells accelerates the absorption of liposomes in the intestine [53].

Orally ingested liposomes form micelles in the presence of phospholipase and bile salts, and then undergo a series of intracellular processes to form chylomicrons. The type of lipid, length and saturation of lipid chains, and the logP value of the loaded drug are potentially useful for forming chylomicrons [54]. Multiple studies in the literature have shown that drugs with logP > 5 and solubility in triglyceride (TG) greater than 50 mg/g are beneficial to the lymphatic transport of chylomicrons [55]. Saturated, monounsaturated, and polyunsaturated lipids differ in their lymphatic transport patterns. In general, lipids with increasing degrees of unsaturation appear to preferentially promote lymphatic lipid transport [56]. Caliph et al. showed that the lymphatic transport of halofantrine (Hf) is highly dependent on the chain length of the co-administered triglyceride lipid. Longer chain lengths lead to higher absorption, suggesting that long-chain phospholipids should be selected to promote lymphatic circulation [57].

## 8. Inhibition of P-gp and CYP3A4

On entry into the small intestinal epithelium, liposomes are subject to P-glycoprotein (P-gp) efflux and CYP3A4 metabolism, which are widely distributed in the intestinal mucosa and hepatocytes—the most evident factors for reduced bioavailability in BCS Class II and IV drugs [58,59].

The synergistic effects of CYP3A4 and P-gp can be avoided by modifying liposomes with a P-gp inhibitor or coating surfaces with a polymer such as Tween 80, Cremophor EL, or vitamin TPGS. All three nonionic surfactants Tween 80, Cremophor EL, and vitamin E TPGS exhibit permeation inhibition of the P-gp-specific substrate R123. The difference is that Vitamin E TPGS reduces the permeability of basolateral-to-apical (BL-AP) permeability, while Tween 80 and Cremophor EL slowly reduce apical-to-basolateral permeability. Gly-sar has been used as a substrate for the peptide transporter hPepT-1, and only Tween 80 reduces Gly-sar permeability in a concentration-dependent manner. Benzoic acid has been used as a substrate for the monocarboxylic acid transporter (MCT); the results showed that only Cremophor EL reduces the transport of benzoic acid. The selection of a suitable nonionic surfactant for mixing into the lipid bilayer could enhance the bioavailability and tissue distribution of the drug [60,61].

Tween 80 markedly represses CYP3A4 protein expression by 70% in human primary hepatocytes (HPH). There are also a large number of CYP3A4 enzymes in small intestinal epithelial cells; they can be mixed into lipid bilayers to delay the metabolism of liposomes by small intestinal cells [62,63]. Nanoparticle components also modulate the enzymatic activity of cytochrome P450, and nanoparticles composed of PLGA (lactide-co-glycolide) have a slight inhibitory effect on CYP2B and CYP3A. The same inhibitory effect has also been observed for inorganic silica nanoparticles; their inhibition shows a size-dependent effect, with 30 nm silica nanoparticles exhibiting a high inhibition level of 73% [63]. Using these characteristics, lipid vesicles can be optimized to minimize the metabolic effects of small intestinal epithelial cells on drugs before their absorption into the blood [64].

## 9. Conclusions

In recent years, there has been a dramatic increase in research on nano-preparations for the oral delivery of therapeutic peptides. Depending on the production processes employed and on the post-processing techniques, liposomes can have different properties. We can choose the appropriate preparation process to form the liposomes we want, such as single unilamellar vesicles (SUVs), large unilamellar vesicles (LUVs), or multilamellar vesicles (MLVs). For post-processing techniques, the rigidity of the bilayer can be enhanced by polymer coating or small molecule compounds that have stronger interactions with phospholipid molecules; this would be suitable for the oral delivery of drugs with fragile chemical structures such as peptides and proteins. The combination of liposomes and other dosage forms to deliver drugs has shown more attractive advantages in gastrointestinal stability, such as the above-mentioned double emulsions and alginate granules, which both contain liposomes.

However, the absorption of liposomes in the small intestinal epithelium is an equally important issue to be taken into account. Liposomes modified by various targets show higher permeability in the intestine; although lipid carriers absorbed into the blood through the small intestine inevitably lose their original membrane structure, the peptides and proteins hidden in the liposomes will face the powerful metabolism of the liver, and so lose their function. Small intestinal lymphatic circulation is undoubtedly the best option available to avoid metabolism that will deactivate the drug. Understanding the processes involved in the oral absorption of liposomes could help in the design of more efficient delivery systems to improve the bioavailability of oral polypeptides. This will be our focus in the future.

## Figures and Tables

**Table 1:** Characteristics of previously reported oral liposomes.

Agent	Phospholipid	Formulation	Property	Modification	EE (%) ± SD	Zeta (mv) ± SD	MD (nm) ± SD	Gain	Ref
Insulin	DMPG (PSC)	Stirring ultrasonic	Anionic phospholipid	-	70.9 ± 2.0	6.2 ± 0.5	29.8 ± 2.3	Degradation of insulin was reduced	[6]
Salmon calcitonin	DPPC DPPE-MCC	TFH + FTC	Amphoteric phospholipid	CS–TGA and CS–TGA–MNA modification	69 ± 12.0	27.9 ± 1.1	604.8 ± 29.6	Reduces blood calcium by 35%	[7]
BSA	SPC	Supercritical assisted process	Amphoteric phospholipid	-	95 ± 3.0	25 ± 5.0	250 ± 58.0	Up to 90% encapsulation rate	[8]
Silymarin (SM)	SPC	Supercritical assisted process	Amphoteric phospholipid	SGC modification	91.4	−62.3	160.50	SM-Lip-SEDS Cmax, AUC increases	[9]
HGH	EPC (GCTE)	DAC	Amphoteric phospholipid	GCTE	31.2 ± 0.5	41.0 ± 1.2	229.7 ± 12.8	3.4% oral bioavailability	[10]
RhIns	PC, DSPE-PEG, Chol	MHF	Amphoteric phospholipid	Chitosan coated with double emulsion carrier	91 ± 4	23 ± 1.0	363 ± 54	The stability and permeability of rhIns increase	[11]
BSA	SPC	RPE	Cationic phospholipid	chitosan coated	44.2 ± 0.3	33.1 ± 0.6	173.7 ± 5.6	More stable	[12]
Calcitonin	DSPC DCP Chol	TFH	Amphoteric phospholipid	Protease inhibitor modified chitosan	>75	39.9 ± 1.6	4460.0	Increases the AAC	[13]
Exendin-4	DOPC DOTAP	RPE	Anionic phospholipid	GCA modified chitosan coating	74.2 ± 2.5	−31 ± 0.2	229 ± 4.0	19% oral bioavailability	[14]
Insulin	SPC	RPE	Amphoteric phospholipid	Biotin-DSPE promotes absorption	-	38.5 ± 3.5	150.0	12% oral bioavailability	[15]
Insulin	SPC	RPE	Amphoteric phospholipid	Thiamine and nicotinic acid Decoration	30.6 ± 2.4	-	125.6 ± 2.9	2.5% oral bioavailability	[16]
Insulin	EPC: Chol DOTAP	TFH	Cationic phospholipid	Protein adsorption	28.7 ± 5.1	−23.1 ± 0.5	164.7 ± 5.3	12% oral bioavailability	[17]
Cy5-amine	POPC POPS,	TFH	Anionic phospholipid	Alginate microcapsule	-	−12.0± 1.0	124 ± 13.0	Longer residence time in the intestine	[18]
Salmon calcitonin	PC	TFH	Amphoteric phospholipid	Bile salt modification	54.9 ± 4.1	-	741 ± 76.9	7.1-times higher bioavailability of sCT	[19]
Insulin	SPC Chol	TFH	Amphoteric phospholipid	FcBP receptor modification	70.9 ± 2.0	6.2 ± 0.5	29.8 ± 2.3	Blood sugar decreased by 47.87%	[20]

TFH, Thin Film Hydration; DAC, dual asymmetric centrifugation; RPE, reversed-phase evaporation; FTC, Freeze–Thaw Cycling; GCTE, tetraether lipid glycerylcaldityl tetraether; DPPE-MCC, 1,2-dipalmitoyl-sn-glycero-3-phosphoethanolamine-N-[4-(p-maleimidomethyl) cyclohexane-carboxamide]; ML, mistletoe lectin; AChE, acetylcholinesterase; SOD, superoxide dismutase; OVA, Ovalbumin; HGH, Human growth hormone; FID, fluorescein isothiocyanate-dextran; rhINS, Recombinant human insulin; DOGS-NTA-Ni, 1,2-Dioleoyl-sn-Glycero-3-N(5-Amino-1-Carboxypentyl)iminodiAcetic Acid]Succinyl (Nickel salt); GCA, glycocholic acid; Biotin, vitamin B7; CS–TGA, chitosan–thioglycolic acid; CS–TGA–MNA, the chitosan-thioglycolic acid 6-mercaptonicotinamide-conjugate;AAC, Area Above Curve; Papp, Apparent Permeability Coefficient; DPPC, 1,2-dipalmitoyl-sn-glycero-3-phosphatidylcholine; POPC, 1-palmitoyl-2-oleoyl-sn-glycero-3-phosphocholine; POPS, 1-palmitoyl-2-oleoyl-sn-glycero-3-phosphoserine; PC, L-α-phosphatidylcholin; SPC, soy phosphatidylcholine; EPC, egg phosphatidylcholine; DOTAP, 1,2-dioleoyl-3-trimethylammoniumpropane; FATP4, The fatty acid transport protein 4; FcBP, Fc domain-binding peptide; DSPE-PEG, 1,2-distearoyl-sn-glycero-3-phosphoethanolamine-N-[biotinyl(polyethylene glycol).

## Data Availability

Data sharing not applicable.

## Abbreviations

EE: encapsulation efficiency; MD, mean diameter; SD, standard deviation; P_app_, Apparent Permeability Coefficients; TEER, transepithelial electrical resistance; API, active pharmaceutical ingredient; SGF, simulated gastric fluid; SIF, simulated intestinal fluid; Ins@MPs, Liposomes embedded in double emulsions; ^99m^Tc-MIBI, sestamibi; Er-lip, Ergosterol modified liposomes; SGC-Liposome, sodium glycopyrrolate modified liposome; Gly-sar, GLYCYL-SARCOSINE.
